# A Lexical T-Maze for Active Inference: A Proof-of-Concept Model of Context Seeking Under Ambiguity

**DOI:** 10.3390/e28101136

**Published:** 2026-10-16

**Authors:** Ahmad Nazzal, Hanada Taha-Thomure

**Affiliations:** ZAI Arabic Research Center, Zayed University, Dubai P.O. Box 19282, United Arab Emirates; hanada.thomure@zu.ac.ae

**Keywords:** active inference, expected free energy, variational free energy, entropy reduction, information seeking, lexical ambiguity, contextual disambiguation, T-maze

## Abstract

Lexical-disambiguation tasks with supplied context do not reveal whether readers would choose to seek evidence before deciding. Therefore, we adapted the spatial T-maze in the active-inference package pymdp to show how agents seek and use contextual information under lexical uncertainty. Agents could choose between two readings or request a contextual hint. We performed two main sets of simulations. First, deterministic simulations used fully informative hints to reproduce context seeking followed by lexical commitment. A hint shifted belief over two readings from equal probabilities (0.50, 0.50) to certainty (1.00, 0.00), reducing entropy by one bit. Second, lexical decision trials were repeated with informative and uninformative hints across 32 paired simulation runs. Agents learned the relationship between the intended reading and the observed hint. Informative experience increased both the probability and simulated frequency of requesting context. This increase persisted when trials ended after one response and feedback. A hint information-gain ablation, in which policies were rescored without crediting the information expected from hints, eliminated the learning-related increase in hint-request probability. These simulations demonstrate how experience changes the expected informational value of context within a lexical task, providing a computational proof of concept for subsequent behavioral experiments.

## 1. Introduction

A written word can support more than one interpretation. For example, the English word (bank) can refer to a financial institution or the side of a river. Context changes which interpretation is likely [1,2]. A reader may seek more evidence before deciding. This can take the form of reading ahead, rereading, or asking for clarification [3,4,5]. Arabic provides another clear example. For instance, the consonantal string كتب (ktb) can be read as كَتَبَ (kataba, “he wrote”) or كُتُب (kutub, “books”), with short-vowel diacritics providing evidence for the correct reading. However, short-vowel diacritics are usually omitted from adult text. This creates uncertainty about the intended reading. In the absence of vowelization, contextual evidence can help readers distinguish between competing readings and reduce uncertainty [6,7,8,9]. Surprisal and entropy have been used to characterize language processing [10,11,12,13,14]. Uncertainty over competing readings can be quantified as entropy [15]. However, entropy does not by itself determine whether to seek more information to resolve uncertainty. That choice is a value-of-information problem [16,17].

In lexical-disambiguation tasks where context is supplied, the final interpretation alone does not reveal whether a reader would have chosen to request that evidence, or how their uncertainty would have influenced that choice. To make this decision explicit, we propose the T-lexical task, a lexical adaptation of the spatial epistemic T-maze used in active inference [18,19,20,21]. The agent can select a reading or request a contextual hint before responding. The task builds on the established spatial T-maze paradigm [22,23,24,25,26,27,28,29,30]. We retain the computational structure and specify its components in lexical terms. The hidden context is the intended reading. The cue is contextual evidence. The two arms are two possible lexical responses. Correct and incorrect feedback replace reward and loss. This follows the broader idea that spatial and cognitive search can share a computational structure [31], while avoiding a claim that lexical processing is itself spatial. The simple design represents prior beliefs explicitly and allows cue reliability to be controlled while context requests, belief updating, and lexical choices are examined within a common task structure.

Information seeking can be modeled through Bayesian value-of-information approaches, reinforcement learning, and active inference [16,17,32]. However, we use active inference because the open-source pymdp package provides an existing T-maze implementation together with established routines for belief updating, policy selection, and learning [33,34]. Adapting this implementation provides a reproducible basis for examining context seeking and lexical choice while retaining an established computational structure. The package implements active inference using partially observable Markov decision process (POMDP) generative models, which represent hidden states, observations, and action-dependent transitions [33]. Variational free energy (VFE) is used during state inference after an observation. Expected free energy (EFE) is used to compare future policies and includes pragmatic and epistemic value [18,19,20,21]. Related active-inference work has addressed communication, listening, and eye movements in reading [35,36,37,38]. For example, existing models simulate conversational questioning [35] or guide eye movements toward informative text [38]. The present adaptation isolates a discrete choice between requesting a contextual hint and committing to one of two lexical readings, allowing context requests and subsequent interpretations to be examined as cue reliability is learned across trials.

In summary, we present numerical studies to simulate how agents solicit contextual information when faced with a lexical decision task under uncertainty. We performed two kinds of simulations. The first (deterministic) simulations considered a simple context in which the cues or hints—available when requested—were (deterministically) informative. This enabled us to reproduce the expected behavior: when the agent was uncertain about the lexical reading, it would ask for a hint before making a decision. In addition, this also allowed us to perform numerical checks of the task implementation.

We then proceeded to the main experiment, in which lexical decision trials were repeated under two conditions: with informative and uninformative hints. Crucially, these numerical analyses included learning of the likelihood mapping between the intended reading and the observed hint. This allowed us to demonstrate information-seeking behavior and how that behavior changes when the agent learns whether the hint is informative. To characterize the underlying imperatives for information seeking, we compared various components of expected free energy between the two conditions. These analyses used multiple replications with stochastic action selection. We were therefore able to reproduce epistemic foraging in this lexical context and show how learning about cue reliability changes the expected informational value of requesting context. This computational proof of concept provides a starting point for behavioral experiments on how readers acquire and use contextual evidence when uncertain.

## 2. Materials and Methods

### 2.1. Study Design and Software

The study used a discrete, factorized partially observable Markov decision process. The implementation used inferactively-pymdp 1.0.4 and its JAX Agent class [33,34]. The notebook contains the model definition, rollouts, learning loop, VFE and EFE calculations, ablations, numerical checks, and figure generation. The analysis had three parts. A deterministic rollout checked the basic mechanism. A paired simulation tested learning across trials. A fixed-parameter rescore separated the utility and information-gain terms in EFE. The submission rerun used Python 3.12.14, JAX and jaxlib 0.4.38, NumPy 1.26.4, Matplotlib 3.9.4, and mediapy 1.2.7.

### 2.2. Mapping the Physical T-Maze to a Lexical Task

**Figure 1:**
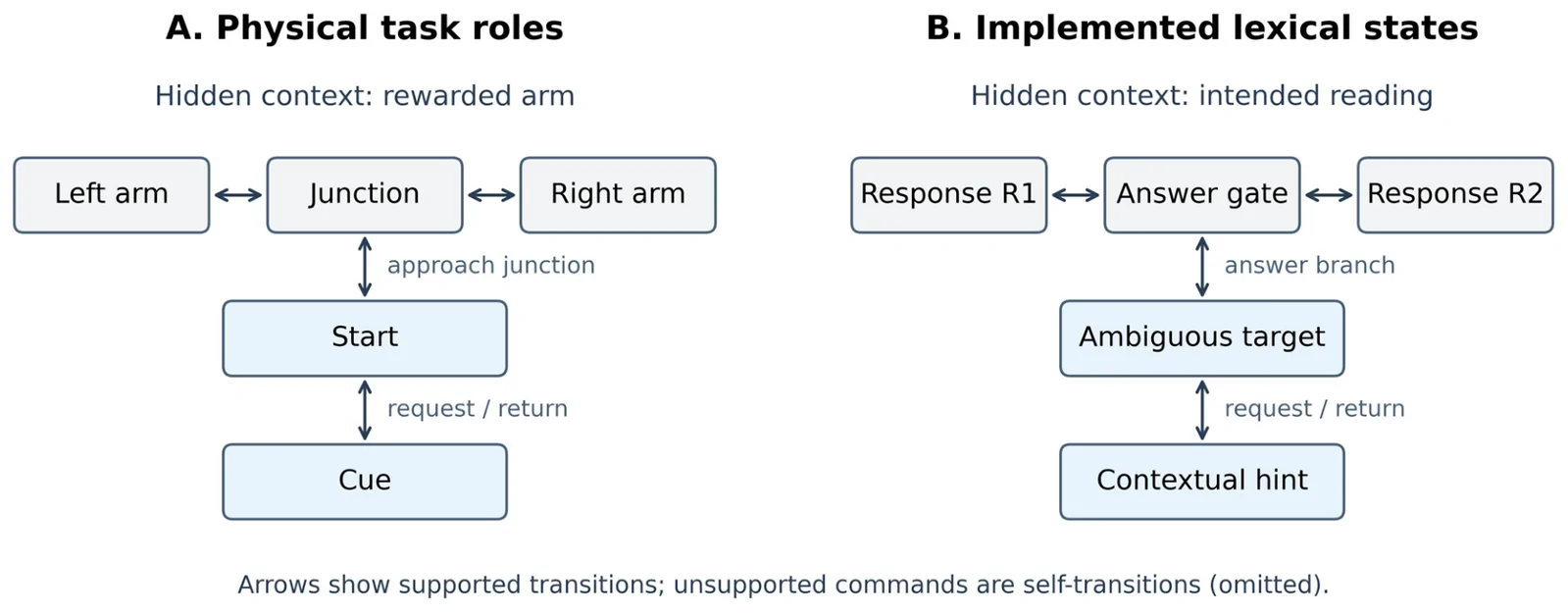
(**A**) The physical T-maze task. (**B**) The lexical T-maze task. Both tasks share one hidden binary state, one optional information source, and two later commitments. The mapping is computational. It does not imply that reading is literally spatial. The answer gate is shown explicitly. Bidirectional arrows represent supported transitions in the retained nonterminal dynamics; unsupported commands cause self-transitions and are omitted.

We adapted the spatial epistemic T-maze by assigning its components lexical roles while retaining their computational relationships. The hidden reward location became the intended reading of an ambiguous word, and the two response arms represented competing lexical interpretations. In the Arabic example, the unvowelized target كتب supported two candidate readings: كَتَبَ (R1) and كُتُب (R2). The agent could request a contextual hint or proceed toward selecting a reading. Visiting the cue therefore corresponded to acquiring contextual evidence, while entering a response arm corresponded to lexical commitment. Correct and incorrect feedback retained the computational roles of preferred and nonpreferred outcomes. This mapping preserved the task’s central decision: whether to seek additional evidence before choosing between two alternatives. Table 1 summarizes the correspondence between physical and lexical elements, and Figure 1 shows the resulting task structure.

### 2.3. Partially Observable Markov Decision Process Specification of Lexical T-Maze

We represented the lexical T-maze as a discrete, factorized, partially observable Markov decision process (POMDP), implemented in pymdp [20,33,34,39]. Partial observability arises because the initial target observation does not identify the intended reading. The agent must infer that reading from the available observations and decide whether to obtain contextual evidence before selecting a response. The Arabic forms provide lexical labels for the model states; the simulated observations are categorical task-stage, hint, and feedback outcomes.

The hidden state was *s_t_* = (*x_t_*, *r*). The task-stage factor *x_t_* contained five states: ambiguous target, response R1, response R2, contextual hint, and answer gate. The reading factor *r* contained the two candidate readings and remained fixed within a trial. The task stage was directly observed, whereas the intended reading had to be inferred. Requesting a hint therefore changed the evidence available to the agent without changing which reading was intended.

The observation was $o_{t}=\left(o_{t}^{x},o_{t}^{f},o_{t}^{h}\right)$, comprising task-stage, feedback, and hint modalities with five, three, and three possible outcomes, respectively. The likelihood mappings *A* specified the probability of each observation conditional on the relevant state factors. The task-stage observation identified the current stage. Feedback indicated no feedback, a correct interpretation, or an incorrect interpretation. The hint modality indicated no hint, support for R1, or support for R2. Cue reliability determined how consistently a hint supported the intended reading. Both hints and response feedback could consequently update beliefs about that reading.

The transition mappings *B* specified how states changed following an action. Under the Markov assumption, the next state depended on the current state and action rather than the full preceding sequence [20,39]. The reading factor remained unchanged, while actions controlled transitions between task stages. Controls were destination-coded: return to target, choose R1, choose R2, request hint, and answer. At the ambiguous target, the two meaningful branches were requesting a hint and proceeding to the answer gate. From the answer gate, a subsequent response command selected R1 or R2. Transitions followed the retained T-maze connectivity shown in Figure 1; unsupported commands produced self-transitions. Nominal action sequences were treated as computational candidates for policy evaluation.

For a policy *π* specifying a sequence of controls *u_t_*, the generative model factorized as:

$$P\left(o_{1:T},s_{1:T}|\pi ,A,B,D\right)=P\left(s_{1}|D\right)\prod_{t=1}^{T}P\left(o_{t}|s_{t},A\right)\prod_{t=1}^{T-1}P\left(s_{t+1}|s_{t},u_{t},B\right)$$

Here, *D* specified the initial state distributions. In the deterministic mechanism check and controlled cue-reliability experiment, each trial began at the ambiguous target with equal prior probabilities for R1 and R2. These reading priors were distinct from the agent’s beliefs about cue reliability, represented in the hint likelihood. In the controlled experiment, the reading priors were reset at each trial, while the learned hint likelihood was retained. Thus, initial uncertainty about the intended reading remained constant across trials, while experience could change the expected usefulness of contextual evidence.

Within a trial, observations updated the agent’s posterior beliefs. The posterior over the intended reading was carried forward when forming the prior for the next observation because the reading itself remained fixed. An informative hint could therefore concentrate belief on one candidate, while subsequent observations could provide further evidence. In the controlled learning experiment, the hint likelihood was updated between trials using the observed cue and the reading posterior obtained from response feedback. The update procedure is specified in Section 2.6.

Preferences *C* assigned values to observations. Correct and incorrect feedback received scores of +2 and −3, respectively, in the deterministic mechanism check, and +3 and −4 in the cue-reliability learning simulation. In the learning simulation, observing the hint position additionally carried a preference score of −0.05. This contribution accumulated for each predicted time step spent at the hint position. Other preference scores were zero. These values were preference scores rather than probabilities. In other words, they are the logarithm of the prior preference probability for each outcome that can be regarded as a prior utility or value. Policy priors *E* were uniform. The resulting policy evaluation and action-selection procedures are described in Section 2.4, Section 2.5 and Section 2.6.

The task retained the original nonterminal T-maze dynamics. Entering a reading-response state did not terminate the rollout or the policy forecast. Subsequent transitions and observations remained possible, including further feedback at response states. Simulations continued for their specified rollout lengths, with the first lexical response used to identify the initial commitment.

### 2.4. Posterior Entropy, VFE, and EFE

The posterior *q_t_*(*r*) represented belief about the intended reading. Its Shannon entropy was [15]:

$$H\left[q_{t}\right]=-\sum_{r}q_{t}\left(r\right)\ln q_{t}\left(r\right).$$

Entropy was reported in nats unless converted to bits. The reduction caused by an observation was:

Δ*H_t_* = *H*[*p_t_*] − *H*[*q_t_*],

where *p_t_* was the empirical prior before that observation.

Posterior VFE was calculated after each observation with calc_vfe. It was checked against its complexity-plus-inaccuracy decomposition:

$$F_{t}=D_{\mathrm{KL}}\left[q_{t}\left(s\right)\;∥\;p_{t}\left(s\right)\right]-E_{q_{t}\left(s\right)}\left[\mathrm{lnP}\left(o_{t}|s\right)\right].$$

VFE is a state-inference diagnostic. It is not a second entropy measure.

Policies were compared by EFE. In the sign convention returned by the software:

$$S\left(\pi \right)=\sum_{\tau }\left[I_{\tau }\left(\pi \right)+U_{\tau }\left(\pi \right)\right],\;G\left(\pi \right)=-S\left(\pi \right),$$

where *U_τ_* is expected preference value and *I_τ_* is expected state information gain. A smaller G is preferred, in the sense that the associated policy is more likely to be selected. The software returns S under the name neg_efe. Fixed action sequences are scored by propagating state beliefs through the transition model, without conditioning future predicted beliefs on hypothetical observations. Policies are rescored after actual observations. The information term sums per-step state information gains, i.e., the expected free energy is a path integral or the accumulated expected free energy over successive time points in the future. The posterior over policies was:

*q*(*π*) = *softmax*[−*γG*(*π*) + *lnE*(*π*)].

State information gain was enabled. Parameter information gain was disabled. The policy prior *E* was uniform. The agent used the package’s fixed-point state-inference routine with 16 iterations. The default marginal action precision was *α* = 1.0.

### 2.5. Deterministic Mechanism Check

The deterministic example used a balanced prior over the two readings, a perfectly diagnostic hint, deterministic feedback, policy horizon 2, and rollout horizon 10. The reported path ended at the first lexical response even though the stored arrays continued to the requested rollout length. The expected sequence was: ambiguous target, contextual hint, return to the target, answer gate, and selected reading. At each time point, the notebook recorded the observation, posterior state belief, empirical prior, VFE, EFE for every policy, posterior policy probability, action, and environment state. The deterministic agent used policy precision γ = 1 and deterministic marginal action selection. Both deterministic examples used seed 0. The hint-first route requires four transitions to reach a reading response, beyond the initial two-step planning horizon; the observed sequence is produced by repeated inference and replanning.

### 2.6. Cue-Reliability Learning Simulation

The main simulation used 32 paired random streams. Each stream completed 30 trials in two conditions. The informative environment produced a reading-consistent hint with probability 0.95. The control condition produced either hint with probability 0.50. Both conditions began with the same agent belief. Its initial conditional cue validity was 0.60. At the hint position, initial concentrations over no hint, support for R1, and support for R2 were (0.05, 2.40, 1.60) for intended R1 and (0.05, 1.60, 2.40) for intended R2. Each column had a total concentration of 4.05. Concentration here corresponds to the concentration counts or parameters of a Dirichlet distribution encoding the likelihood of an observation, given some hidden state or cause. These concentration parameters are accumulated on the basis of experience to simulate learning. This means that confidence in the likelihood mappings or contingencies is scored by the total number of concentration parameters or Dirichlet counts, which increases with learning, as we will see below. Column normalization gave the likelihood means, and the small null-hint probability was retained in the mutual-information calculation. Each trial began with equal prior probability for R1 and R2. The model used a four-step policy horizon, policy precision *γ* = 4, and stochastic marginal action sampling. The nominal policy set contained 5^4^ = 625 four-action strings. Of these, 125 began with request_hint and 125 began with answer. Within-trial likelihood and transition learning were disabled. When a trial contained both a cue and feedback, the first cue outcome was associated with the reading posterior at the first feedback time. In all update-eligible trials, the first cue preceded the first feedback. The hint likelihood concentration was updated once after the trial:

$$pA^{h}\left(o^{h},x_{\mathrm{hint}},r\right)\leftarrow pA^{h}\left(o^{h},x_{\mathrm{hint}},r\right)+\eta \;1\left[o^{h}=o_{\mathrm{observed}}\right]\;q_{t_{f}}\left(r\right),\;\eta =1.$$

Here, the subscripted time denotes the first feedback observation, and the observed cue in the indicator is the first cue outcome. Trials without both observations did not update the cue likelihood. A new agent was then created for the next trial with the updated concentrations. Parameter information gain was disabled. The agent therefore requested a cue for the current lexical decision, not because it prospectively valued learning the cue parameter. At the first decision of each trial, we recorded estimated cue validity, posterior mass on hint-first policies, and the sampled first action. We summarized trials 1–5 as the early window and trials 26–30 as the late window. The paired experiment used seed 2026 and num_timesteps = 7, which stores eight observation and inference states at times 0 through 7. First-action outcomes include every trial; feedback-grounded learning requires both observations within that window.

The two conditions shared random streams. Change was summarized as:

(informative late − informative early) − (control late − control early).

### 2.7. EFE Decomposition and Cue-Information Ablation

Initial and learned hint likelihoods were frozen and rescored from the same starting beliefs. The score was separated into expected utility and state information gain. Information gain was also separated by observation modality. The information expected from one hint was calculated as mutual information under the agent’s retained hint likelihood $\hat{A}$ and a balanced reading prior:

$$I_{\hat{A}}\left(R;O^{h}\right)=\sum_{r,o}q\left(r\right)\hat{A}\left(o|r\right)\ln\frac{\hat{A}\left(o|r\right)}{\sum_{r^{\prime }}q\left(r^{\prime }\right)\hat{A}\left(o|r^{\prime }\right)}$$

The key ablation removed only contextual-hint information gain from policy scores. The learned likelihood, preferences, feedback information, policy set, precision, and starting beliefs were left unchanged. If the learning-related policy shift depends on the informational value of the cue, that shift should disappear under this ablation. This was a fixed-state policy rescore, not a new behavioral rollout under ablation. Main paired-stream change contrasts in this analysis used 5000 bootstrap resamples.

### 2.8. Robustness Analysis with an Explicit Stopping Rule

We repeated the cue-learning experiment to test whether the findings depended on continuing the task after a lexical response. In the first variant, each trial ended after one response and its feedback. In the second variant, only one contextual hint could be obtained before responding. All other experimental settings were retained. We compared both variants with the original task, examining changes in hint-request probabilities and simulated hint requests as cue reliability was learned.

## 3. Results

### 3.1. The Lexical T-Maze Produced the Intended Sequence

**Figure 2:**
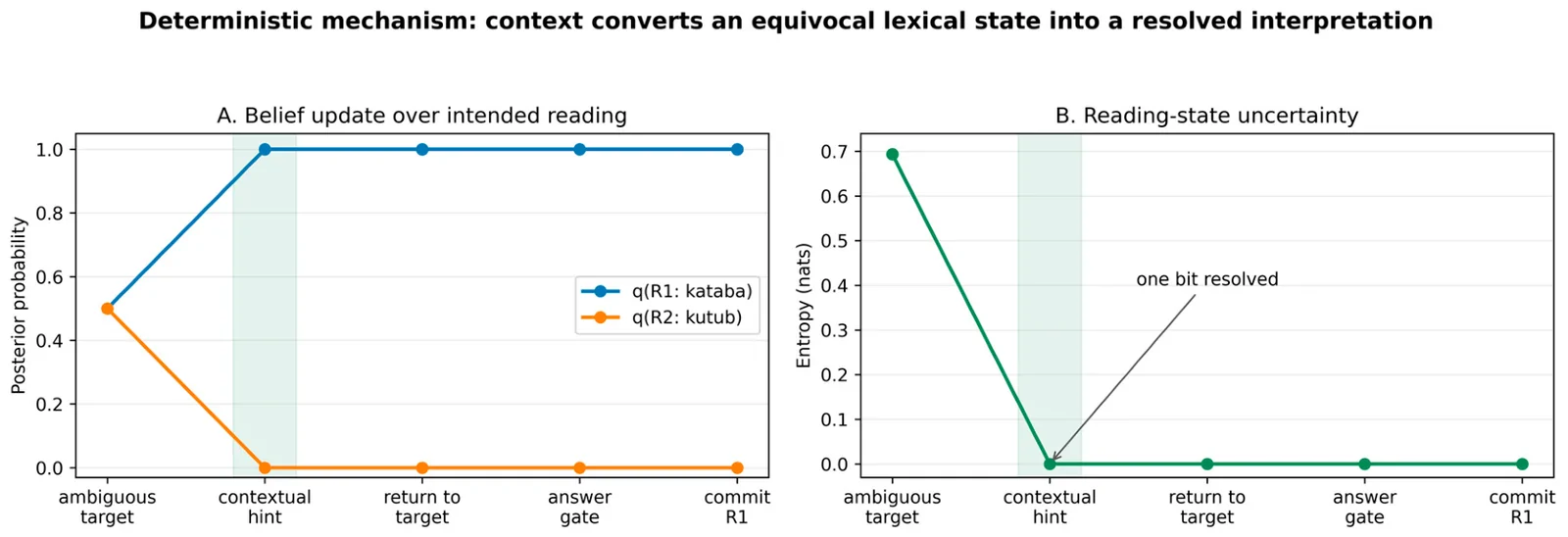
(**A**) Belief update over intended reading. Diagnostic contextual hint changed a uniform posterior over two readings into certainty. (**B**) Reading-state uncertainty. The entropy reduction was one bit. The pale green shaded band marks the contextual-hint stage, when the agent receives the hint and updates its beliefs.

The representative deterministic rollout began at كتب with *q*(*R*1, *R*2) = (0.50, 0.50). It selected request_hint. The perfectly diagnostic hint supported R1 and changed the posterior to (1.00, 0.00). The model then returned to the target, entered the answer gate, selected R1 (كَتَبَ, kataba), and received correct feedback. Posterior entropy fell from ln2 = 0.6931 nats to 0. This is a reduction of one bit. The posterior remained resolved through commitment (Figure 2). 

### 3.2. VFE and EFE Behaved as Separate Quantities

We calculated VFE after each observed outcome. VFE was approximately 0 at the initial state. It rose to 0.6931 nats when the diagnostic cue was assimilated and returned to approximately 0 at the later stages. This peak reflects the posterior update under the model. EFE was calculated prospectively for each policy. Table 2 shows three summaries over the first five stages. EFE need not decrease at every stage. It is recalculated from the current position and beliefs.

These values confirm that we scored uncertainty, posterior inference, and prospective policy separately.

### 3.3. Informative Experience Increased Later Hint Seeking

**Figure 3:**
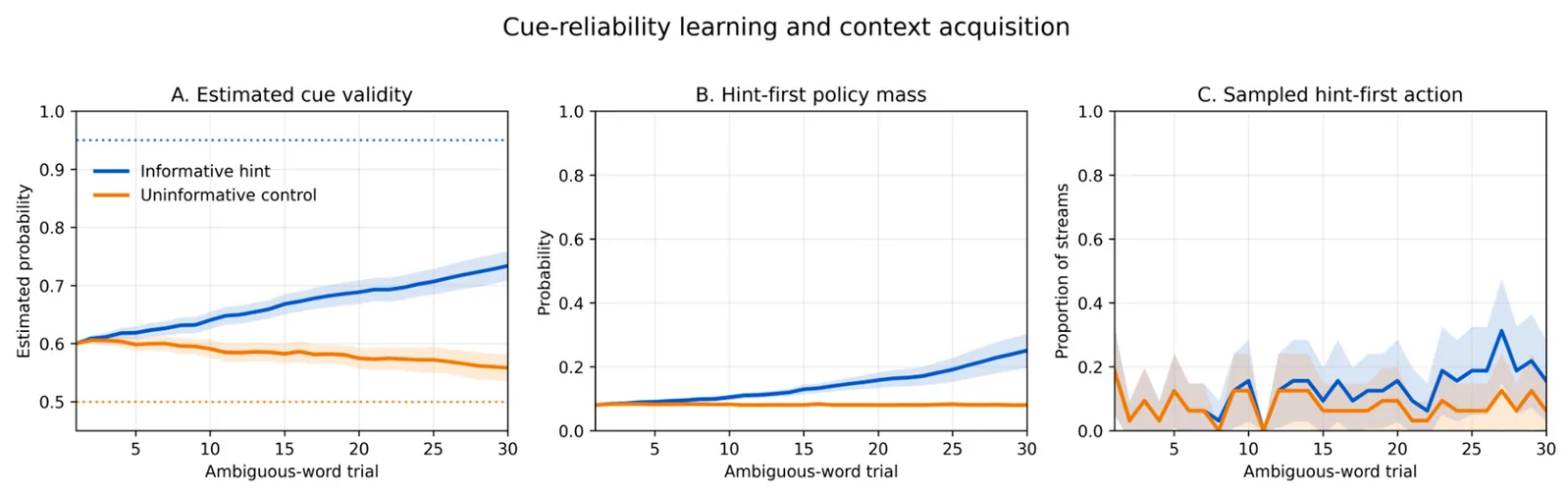
Learning trajectories from the paired simulation. Panel (**A**) show estimated cue validity. Panel (**B**) show posterior hint-policy mass. Panel (**C**) show the sampled first action. Lines show means across streams. Ribbons show mean ± 1.96 SEM. The 32 streams are simulated agents. Dotted lines in the validity panel mark the environmental values, 0.95 and 0.50.

The informative and control conditions began with the same cue model. Their later behavior differed. In the informative condition, mean estimated validity increased from 0.611 in the early window to 0.723 in the late window. In the control condition, it fell from 0.603 to 0.563. Posterior mass on hint-first policies increased from 0.086 to 0.228 in the informative condition. It changed from 0.083 to 0.081 in the control. The sampled hint-first rate increased from 0.094 to 0.213 under informative cues and from 0.094 to 0.088 in the control (Table 3). Figure 3 shows the learning trajectories across trials. 

The informative streams received 6.59 cue-feedback updates on average. Control streams received 3.88. Cue exposure therefore co-evolved with policy selection. The result illustrates the intended learning of how informative the hint is, in a given context, illustrating the autodidactic nature of active inference and learning in this setting.

### 3.4. Cue Information Gain Explained the Policy Shift

**Figure 4:**
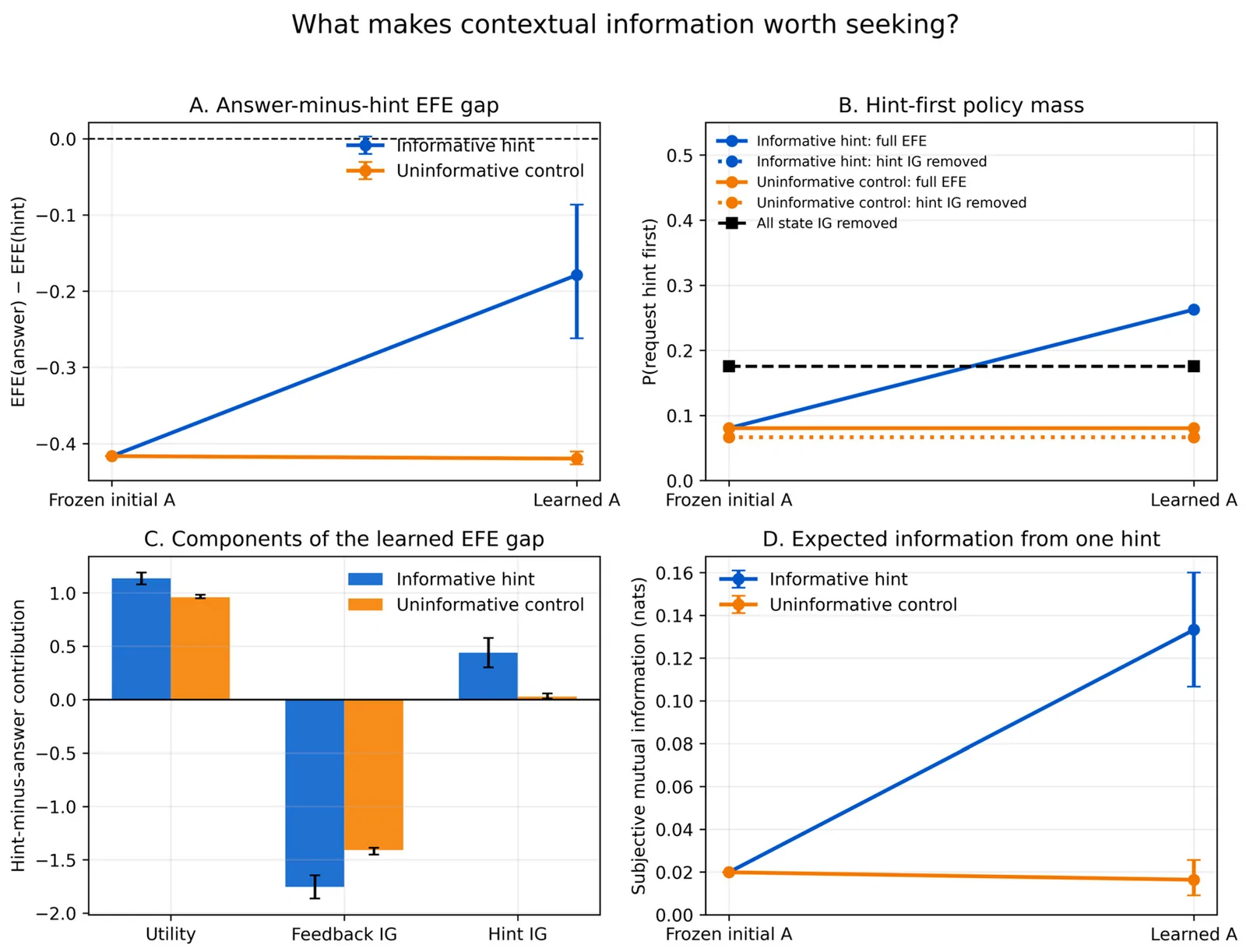
Fixed-state expected free energy (EFE) decomposition and cue-information ablation. (**A**) EFE difference between the best answer-first and hint-first policies, calculated as G(answer) − G(hint); positive values favor requesting a hint first, whereas negative values favor answering first. (**B**) Total posterior probability of hint-first policies under full EFE scoring, after removing hint information gain (IG), and after removing all state IG. Removing hint IG eliminated the learning-related increase in hint-first policy probability. The blue and orange dotted curves overlap, with the orange curve covering the blue curve; the black dashed curve represents removal of all state IG. (**C**) Hint-minus-answer differences in expected utility, feedback IG, and hint IG after learning. These components use the same policy pair selected under full EFE and sum to the EFE gap in panel (**A**). (**D**) Expected information from one hint, expressed as mutual information in nats. Blue and orange represent the informative and uninformative conditions, respectively. Initial and learned hint likelihoods were rescored from the same balanced reading prior. IG denotes state information gain. Error bars in panels (**A**,**C**,**D**) show 95% percentile bootstrap intervals from 5000 resamples of 32 simulation streams; panel (**B**) shows means without error bars.

Figure 4 summarizes the EFE decomposition, cue-information ablation, and changes in the expected information supplied by a contextual hint. The information expected from one hint was 0.0199 nats before learning. It increased to 0.1333 nats after informative learning. It was 0.0164 nats after control learning. The paired informative-minus-control change was 0.117 nats, with a 95% paired-stream bootstrap interval of [0.089, 0.146].

The full EFE comparison gave the same pattern. The signed gap *G*(*answer*) − *G*(*hint*) changed from −0.416 initially to −0.179 after informative learning. It was −0.420 after control learning. The mean gap remained negative: the best answer-first policy retained lower EFE on average across streams under the focal horizon and preferences. In sum, 7 of the 32 informative learned models favored the best hint-first policy, whereas 25 favored the best answer-first policy. Informative learning nevertheless made hint-first policies more competitive.

Raw posterior mass on hint-first policies rose from 0.081 to 0.263 after informative learning. It remained 0.080 after control learning. When only contextual-hint information gain was removed, this posterior mass was 0.066 at all checkpoints. Within the two meaningful first branches, *P*(*hint*∣*hint* or *answer*) rose from 0.141 to 0.421 after informative learning. It remained 0.141 in control and 0.116 under the cue-information ablation.

Table 4 reports means across 32 streams. For each stream, the best hint-first and answer-first policies were selected under the full score; all component differences use that same pair. The policy-mass column instead sums posterior probability over all 125 hint-first policies. The components sum to the full EFE gap for the selected pair. The full EFE gap, calculated as *G*_answer_ − *G*_hint_, compares the best answer-first and hint-first policies using both expected utility and expected information gain; positive values favor requesting a hint first, whereas negative values favor answering first.

### 3.5. Results Under the Stopping Rule

The learning-related increase in context seeking persisted when trials ended after one response and its feedback. With informative hints, the model’s hint-request probability increased from 0.131 in early trials to 0.314 in late trials, while remaining approximately stable with uninformative hints. Simulated hint requests also increased, from 13.8% to 29.4%.

When only one hint was available per trial, context seeking still occurred, but the learning-related change was smaller. Hint-request probability increased from 0.112 to 0.137, while simulated requests remained at 12.5% in both early and late trials. Both stopping variants produced fewer completed responses within the original trial limit.

These comparisons identify cue availability and trial termination as task-design choices that influence the expression of context seeking and should be specified when developing behavioral versions of the lexical T-maze. Full comparisons are provided in the Supplementary File S3.

## 4. Discussion

### 4.1. Main Finding

We mapped a spatial epistemic T-maze to a lexical ambiguity task without changing its core decision structure. The model began uncertain. It requested a hint. The hint reduced uncertainty about the lexical context and the model then committed to the supported lexical reading. VFE tracked posterior inference after observations. EFE compared future policies before actions. The learning simulation adds one step. Repeated informative experience changed the agent’s cue model. This increased the expected information supplied by a hint resulting in the agent learning to request hint more often. Removing only the hint information-gain term in a fixed-state rescore eliminated the learning-related increase in hint-first policy mass. The learning-related increase in context seeking persisted in the additional stopping analysis.

### 4.2. What the Study Contributes

The contribution of this study is a reproducible lexical context-seeking task implemented in pymdp [33,34]. Conventional lexical-disambiguation systems generally identify an interpretation from supplied context [40,41]. The lexical T-maze represents the decision to request contextual evidence before selecting an interpretation. It provides an explicit mapping from competing readings, contextual hints, and response feedback to the states, observations, and actions of an established active-inference task.

Bayesian decision-making, value-of-information, reinforcement-learning, and active-inference approaches address the decision to acquire information [16,17,32,42]. Active inference and reinforcement learning can both be applied to decision problems formulated as POMDPs [32]. A computational comparison between alternative models was outside the scope of the current study. For context, a recent empirical comparison of active-inference and reinforcement-learning models fitted to human choices in a three-armed bandit task favored active inference in model comparison, although choice-prediction accuracy was similar and the models provided partially distinct explanations of behavior [43]. Pymdp itself implements active inference using POMDP generative models [33]. The present adaptation retains this computational foundation and makes its application to a lexical task explicit and executable. The novelty of this study lies in adapting the exploration–exploitation structure of the spatial epistemic T-maze to lexical tasks in which an agent can request contextual evidence before committing to an interpretation.

In a corresponding experiment, participants could choose between two readings or request additional context before responding. This would allow researchers to test when readers seek further evidence and how that evidence influences their eventual interpretation. The adaptation also makes explicit how prior beliefs over competing lexical readings are combined with contextual evidence to form posterior beliefs [20,39]. In the controlled cue-reliability experiment, each trial begins with equal prior probabilities over the two readings, while the learned cue likelihood is retained across trials. This separates initial uncertainty about the intended reading from experience-dependent changes in the expected reliability of contextual evidence. In human behavioral studies, priors could be estimated separately by surveying readers of a language or by using language statistics derived from large language models. The task therefore provides a basis for future behavioral testing of the interaction between prior expectations, evidence acquisition, and belief revision in a lexical task.

### 4.3. Scope and Limitations

This study provides a practical adaptation of the established epistemic T-maze to the lexical domain, implemented in pymdp as a reproducible computational proof of concept. Its scope is to provide a working task structure in which contextual evidence can be requested before selecting between competing lexical readings. The Arabic forms serve as illustrative labels for two alternatives. The model does not process orthography, morphology, or sentence meaning. Initial beliefs, preferences, and task contingencies were specified by the modeler, while cue likelihoods were updated through simulated experience. The simulations establish the operation of this task under controlled conditions; correspondence with human reading behavior remains to be evaluated.

The original implementation retained nonterminal T-maze dynamics. The additional stopping analysis showed that the learning-related increase in context seeking persisted when trials ended after one response and its feedback. When contextual evidence was restricted to one hint per trial, context seeking remained present, although the increase in hint-request probability was smaller and sampled requests did not increase between early and late trials. These comparisons demonstrate how cue availability and trial termination influence the expression of context seeking within the implemented task and inform its subsequent behavioral use.

The adaptation therefore provides a reproducible starting point for developing lexical experiments with optional contextual evidence. Application to a particular experiment requires specifying its stimuli, cue availability, response options, and stopping rules, followed by fitting and evaluating the model against participant data [39,44].

### 4.4. Next Step

The next step is to collect behavioral data and fit an active-inference model. The empirical test can remain simple. Participants could see an ambiguous lexical target and choose either to answer or reveal a short context. Cue validity and request cost could be varied. The main outcome would be whether context requests increase when cues are more reliable and decrease when they are more costly. Eye tracking could measure fixation duration, rereading, and transitions between the target and its context. EEG could provide time-resolved measures of neural responses to ambiguity, cue presentation, belief updating, and lexical commitment. These measures would allow the model’s predicted sequence of uncertainty, information seeking, updating, and commitment to be compared with behavior and neural dynamics.

## 5. Conclusions

We adapted the spatial epistemic T-maze in pymdp to represent a choice between requesting contextual evidence and selecting a lexical reading. A diagnostic hint resolved uncertainty between two readings in the deterministic example. Across repeated trials, learning cue reliability changed the expected informational value of context and the tendency to request it. Removing the cue-information term during policy rescoring eliminated the learning-related increase in hint-request probability. This increase persisted when trials ended after one response and its feedback.

The contribution is a reproducible adaptation of an established task to the lexical domain and a computational proof of concept. It makes context seeking an explicit choice within a simple lexical task and provides an executable starting point for behavioral experiments on how readers acquire and use contextual evidence under uncertainty. Its broader theoretical value and behavioral relevance remain to be established through comparison with alternative models and empirical data.

## Figures and Tables

**Table 1:** Role-preserving mapping from the physical T-maze to the lexical task.

Physical T-Maze	Computational Role	Lexical T-Maze
Hidden reward location	Unobserved binary context	Intended reading: R1 or R2
Start or junction	Initial decision state	Ambiguous unvowelized target كتب
Cue location	Optional informative observation	Contextual hint supporting R1 or R2
Travel to cue	Information-seeking action	Request context before answering
Left or right arm	Mutually exclusive commitment	Choose R1: كَتَبَ or R2: كُتُب
Reward or loss	Preferred or nonpreferred outcome	Correct or incorrect feedback

**Table 2:** Mean deterministic-rollout diagnostics across nine streams over the first five stages. The final stage is commitment to the correct reading, R1 or R2. All information quantities are in nats. MAP denotes the maximum a posteriori policy.

Stage	Posterior Entropy	Posterior VFE	Posterior-Mean EFE	MAP-Policy EFE
Ambiguous target	0.6931	approximately 0	−0.6806	−1.3863
Contextual hint	0	0.6931	approximately 0	0
Return to target	0	approximately 0	−0.4806	−2.0000
Answer gate	0	approximately 0	−3.6445	−4.0000
Correct-reading commitment	0	approximately 0	−3.9056	−4.0000

**Table 3:** Early-to-late learning summaries.

Measure	Informative Early	Informative Late	Control Early	Control Late	Paired Change Contrast [95% Bootstrap Interval]
Estimated hint validity	0.611	0.723	0.603	0.563	0.152 [0.121, 0.183]
Posterior mass on hint-first policies	0.086	0.228	0.083	0.081	0.144 [0.101, 0.192]
Sampled hint-first action rate	0.094	0.213	0.094	0.088	0.125 [0.069, 0.188]

**Table 4:** Fixed-state EFE decomposition for the best hint-first and answer-first policies.

Condition and Likelihood	Full EFE Gap	Utility Term	Feedback Information Term	Hint Information Term	*p* (Hint First)
Informative: initial	−0.416	0.950	−1.386	0.020	0.081
Informative: learned	−0.179	1.136	−1.755	0.440	0.263
Control: initial	−0.416	0.950	−1.386	0.020	0.081
Control: learned	−0.420	0.961	−1.408	0.027	0.080

## Data Availability

The executable notebook, pinned software environment, random seeds, generated outputs, and figure-generation code are provided in Supplementary Files S1–S3. No human-participant data were collected.

## Supplementary Materials

The following supporting information can be downloaded at: https://www.mdpi.com/article/10.3390/e28101136/s1, File S1 is Lexical_TMaze_Proof_of_Concept_reproducible.ipynb. It contains the deterministic verification, stochastic rollouts, cue-reliability learning simulation, posterior entropy analysis, VFE calculation, EFE calculation and decomposition, cue-information ablation, sensitivity grid, numerical assertions, and figure-generation code. File S2, Lexical_TMaze_supporting_files.zip, provides the README, resolved environment, execution script, figure sources and exports, and numerical arrays. Main exports match Figure 1, Figure 2, Figure 3 and Figure 4; the condition-congruent probe and validity-by-penalty surface are Supplementary Figures S1 and S2. File S3, T_lexical_Stopping_Robustness.ipynb, provides the executable notebook and results comparing the original task with two stopping variants: termination after one response and feedback, and the same stopping rule with access restricted to one contextual hint per trial. File S3 also includes a joint-information sensitivity analysis in which fixed policies are rescored using frozen models, accounting for overlap between information supplied by hints and feedback about the intended reading. This analysis examines the resulting changes in hint-request probabilities and policy rankings.
